# Carbohydrate Rinse Fails to Enhance Cycling Performance or Alter Metabolic and Autonomic Recovery in Recreational Cyclists

**DOI:** 10.2478/hukin-2022-0036

**Published:** 2022-09-08

**Authors:** Cassie M. Williamson-Reisdorph, Emily E. Bechke, Cherilyn McLester, Robert Buresh, Melinda Millard-Stafford, Zackery Green, Rasmus Rooks, Brett Nickerson, Brian M. Kliszczewicz

**Affiliations:** 1 School of Integrative Physiology and Athletic Training, University of Montana, Missoula, MT 59801, USA; 2 Department of Exercise Science and Sport Management, Kennesaw State University, Kennesaw, GA 30144, USA; 3 School of Biological Sciences, Georgia Institute of Technology, Atlanta, GA 30332, USA; 4 College of Nursing and Health Sciences, Texas A&M International University, Laredo, TX 78041, USA

**Keywords:** carbohydrate solution, endurance performance, cycling, heart rate variability, autonomic nervous system, exercise metabolism

## Abstract

The purpose of the study was to examine the effects of carbohydrate (CHO) mouth rinsing on autonomic and metabolic recovery as well as cycling performance. Ten male recreational cyclists (age = 30 ± 6 years, VO_2peak_ = 54.5 ± 8.1 mL·kg^-1^·min^-1^) completed a randomized, double-blind, placebo-controlled, crossover designed study. A CHO or a placebo (PLA) rinse was administered every 12.5% of a work to completion trial (75%W_max_). Heart rate variability (lnRMSSD), the respiratory exchange ratio, and plasma epinephrine, norepinephrine, insulin, glucose, free fatty acids (FFA), and lactate were measured pre- and post-exercise. The CHO rinse did not improve time to completion of the test trial (CHO: 4108 ± 307 s, PLA: 4176 ± 374 s, p = 0.545). Further, the CHO rinse did not impact autonomic recovery, as measured by lnRMSSD (p = 0.787) and epinephrine (p = 0.132). Metabolic biomarkers were also unaffected by the CHO rinse, with no differences observed in responses of FFA (p = 0.064), lactate (p = 0.302), glucose (p = 0.113) or insulin (p = 0.408). Therefore, the CHO mouth rinse does not reduce the acute sympathetic response following strenuous exercise and does not result in improvements in cycling time to completion.

## Introduction

The use of a carbohydrate (CHO) mouth rinse as an ergogenic aid has become a considerable interest in sports nutrition research (Jeukendrup et al., 2013; Pottier et al., 2010; Rollo et al., 2010). Compared to traditional CHO drink ingestion, rinsing protocols may provide comparable performance improvements during endurance exercise (Pottier et al., 2010). A landmark study conducted by Carter et al. (2004) demonstrated a CHO rinse (6.4% maltodextrin solution) elicited a 2.9% improvement in a cycling trial of endurance trained cyclists. These findings have led to an increased popularity of CHO rinsing as a supplementation strategy in endurance sports, especially in those in which athletes experience gastrointestinal distress with CHO ingestion (Jeukendrup et al., 2013). However, several follow-up studies reported equivocal findings in performance improvements using a CHO rinse, with most positive findings occurring in endurance exercise of approximately one-hour in duration (Carter et al., 2004, 2016; James et al., 2017; Pottier et al., 2010). Furthermore, whether CHO rinsing provides residual benefits in subsequent exercise bouts or recovery from exercise is currently unknown. Specifically, the acute exercise recovery period is important in athletics and competition due to its influence on an athlete’s overall recovery status and readiness to perform (Stanley et al., 2013).

Currently, the physiological mechanisms by which a CHO rinse improves performance are not entirely clear. One proposed mechanism is that CHO in the rinse acts upon receptors in the oral cavity, stimulating brain regions associated with rewarding stimuli (Chambers et al., 2009; Jeukendrup et al., 2013). The initiation of a motor command from the brain mediates autonomic nervous system (ANS) activity, alters central command, and may thereby influence physiological responses and attenuate fatigue (Chambers et al., 2009). The potential alterations of ANS activity could impact metabolic fluctuation of lipolysis and glycogenolysis through heightened sympathoadrenal activity (Farrell et al., 2011; Lafontan and Langin, 2009).

Research on the metabolic and performance effects of CHO rinsing during a bout of exercise has been conducted; however, the autonomic effects extending into the recovery period immediately following exercise remain unexplored. HRV has been utilized recently to assess recovery from exercise (Gifford et al., 2018). Moreno et al. (2013) evaluated HRV recovery following a 60-min exercise protocol and found that autonomic modulation was improved in those who ingested the CHO mixed beverage, leading to the notion that in some way, the presence of CHO improves ANS recovery. In order to determine if and when rinsing is a beneficial strategy for athletes, the physiological responses to CHO rinsing during exercise recovery may, therefore, be of particular interest for those who perform frequent or multiple bouts with limited time for recovery. Therefore, the purpose of the study was to examine the effects of CHO rinsing on autonomic and metabolic responses during the acute exercise recovery period to determine whether rinsing could enhance performance and post-exercise recovery. We hypothesized that CHO rinsing would lead to enhanced recovery through improved parasympathetic rebound of the ANS following exercise, which would ultimately increase the rate of return to metabolic homeostasis. Recovery was assessed via autonomic and metabolic activity with heart rate variability (HRV), plasma catecholamine concentrations, respiratory exchange ratio (RER), insulin (INS), glucose (GLU), lactate (LA^-^), and free fatty acids (FFA).

## Methods

### Participants

Twenty male recreational cyclists attempted the protocol, and 10 completed it (age = 30.6 ± 6.5 years, body mass = 80.6 ± 8.5 kg, VO_2peak_ = 54.5 ± 8.1 mL·kg^-1^·min^-1^). Participants’ characteristics can be seen in Table 1. Written informed consent was obtained prior to participation in the study, which was approved by the University Institutional Review Board and followed the guidelines of the Declaration of Helsinki (Association, 2014). Participants were classified as having a minimum of “good” cardiorespiratory fitness levels as determined by VO_2max_ testing (Thompson et al., 2010). The inclusion criteria required that each participant trained at least twice per week for a minimum of one-hour each session. Those who reported having any orthopedic problems or were suffering from any cardiovascular, pulmonary, or metabolic diseases, as determined through a health history questionnaire were excluded from the study.

**Table 1:** Participants’ Characteristics

Characteristic	Values ± SD
Age (yrs)	30.6 ± 6.5

Height (cm)	180 ± 5.4
Body mass (kg)	80.6 ± 8.5
Body Fat (%)	17.7 ± 4.5
W_max_ (W)	353.1 ± 8.1
VO_2max_ (mL·kg^-1^·min^-1^)	54.4 ± 8.1

*N = 10; Values presented as mean ± *SD*.

### Design and Procedures

**Figure 1:**
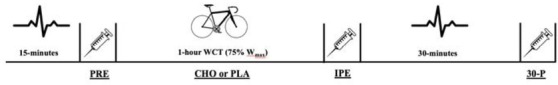
Experimental Design

The study utilized a double-blind, placebo-controlled, randomized crossover design with subjects serving as their own control. Data remained blinded until all statistical operations were completed. The experimental test protocol can be seen in Figure 1. All data collection was performed in the Exercise Physiology Laboratory at the same time of day, between the hours of 5 and 10 AM, on three separate occasions with three to nine days between visits. The initial visit consisted of a review of the procedures, signing of the informed consent, completion of the health history questionnaire, anthropometric measurements, a VO_2max_ test, and a five-minute familiarization period. The remaining two trials were performed with either the CHO or placebo (PLA) solutions being administered every 12.5% of work completed (8 total rinses) during the trial. Pre- and post-exercise measurements of HRV were collected through a heart rate monitor and RER measurements were collected with a metabolic cart. Concentrations of E, NE, INS, and FFA were obtained through blood sampling and LA-, GLU, hemoglobin, and hematocrit were measured via capillary puncture at pre-exercise (PRE), immediately post-exercise (IPE) and 30-min post-exercise (30-P). After baseline samples, participants engaged in a 5-min warm-up followed by the estimated 1-hour cycling work to completion trial (WCT) at the prescribed workloads. After the completion of the bout, designated blood draws, HRV markers, and RER were collected during a 30-min recovery period.

#### Preliminary Testing (Anthropometric Measurements & VO_2max_ Testing) & Familiarization Period

Participants were required to fast for a period of 10-hours, abstain from alcohol and exercise for 24-hours, and avoid caffeine for 12-hours before testing sessions. During the first visit, anthropometric measurements were collected, and body composition was assessed using dual-energy x-ray absorptiometry (GE Lunar iDXA, Boston, MA).

The W_max_ test, as described by Kuipers et al. (1985), was performed on a cycle ergometer (Lode Excalibur Sport, LEM 9.4.4.0, Groningen, Netherlands) to determine maximal workload (W_max_), VO_2max_, and maximal heart rate (HR_max_). The cycle ergometer was adjusted to the participants desired fit and the position was measured and repeated for all remaining visits. Participants began with a 5-min warm-up at 100 watts. Following the warm-up, the workload was increased by 50 watts every 2.5 minutes until the heart rate reached 160 beats per minute with a pedal rate which was maintained at ≥ 60 rpm. The workload was then increased by 25 watts every 2.5 minutes until the participant achieved volitional exhaustion. Expired gas fractions were sampled using a metabolic cart. Following the maximal exercise test which occurred during the first visit, participants were given a recovery period of self-selected duration. The cycle ergometer was then set to a linear mode based on the individual’s calculated workload and participants were asked to pedal at the prescribed resistance for a period of 5 minutes in order to familiarize them with the linear mode of the ergometer.

W_max_ for the one hour WCT was calculated according to the established protocol of Jeukendrup et al. (1996). Following this calculation, the total work for one hour was calculated (the average W_max_ of participants was 353 ± 8.1) based on the following equations (Jeukendrup et al., 1996), which were equivalent to 75% W_max_:

### Total Work for the 1‐hour WCT (J) = 0.75 x W_max_ x 3600

The cycle ergometer was set to the linear mode so that 75% W_max_ would be obtained within one hour if participants pedaled at their preferred pedal rate for the entirety of the trial. In order to set the cycle ergometer to the linear mode during the WCT, the participants’ linear factor (L) was calculated using the equation W = L x (RPM)^2^, with the RPM being equivalent to their preferred pedal rate, which was determined during the VO_2max_ test. Participants were not required to maintain this rate and variations in time to completion (i.e., > 1-hour or < 1-hour) occurred. The average preferred pedal rate of cyclists within this study was 82 ± 5.8 RPM and the calculated linear factor was 0.04 ± 0.01.

#### Experimental Trials

Participants kept a nutritional log for the 24-hour period leading up to their second visit and were asked to repeat these meals between trials. Upon arrival, participants were fitted with a heart rate monitor (Polar Team^2^, Bethpage, NY) to evaluate HRV and an oxygen consumption mask to measure RER via the metabolic cart (Parvomedics TrueOne^®^ 2400, Sandy, UT), and asked to sit quietly in a dimly lit room for a 15-min period. Following the resting period, LA-, GLU, hematocrit, and hemoglobin were measured via capillary puncture and a 6 mL pre-exercise blood sample was taken.

Participants completed a standardized warm-up of 5 min with 100 watts at the WCT selected pedal rate (Jeukendrup et al., 1996). The ergometer then transitioned to the 1-hour WCT in the linear mode at their calculated workloads. Participants were instructed to complete the work as quickly as possible and they were provided with the same cycling simulation video without sound. After every 12.5% of the total work completed, the participants’ HR and RPE were recorded (8 total time points). Following the WCT, HRV and RER were monitored during the 30-min seated recovery period and blood samples were taken at designated time points (IPE and 30-P).

#### Carbohydrate Rinse Protocol

**Figure 2:**
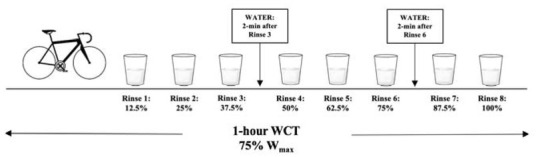
Carbohydrate and Placebo Rinse Protocol

Participants rinsed their mouths with either a commercially available CHO drink (Powerade^®^, Fruit Punch) or an artificially sweetened beverage as the placebo (Powerade Zero^®^, Kiwi Pineapple) in randomized order. In order to increase the difficulty for identifying the artificial sweetener, we selected two different flavors of sports drink. Commercially available drinks have been utilized in previous studies and demonstrated significant improvements in time to completion (Pottier et al., 2010). The concentration of the CHO solution selected for this study was 6.4%, which has been shown to improve performance (Carter et al., 2004; Chambers et al., 2009; Pottier et al., 2010) and diminished response to higher concentrations (Devenney et al., 2016; James et al., 2017). Artificial sweeteners have been used as a control solution in previous studies (Kumar et al., 2016; Pottier et al., 2010). A 25 mL bolus was measured into a cup and rinsed around the mouth following the completion of each 12.5% work segment and expectorated into a container. Rinse duration of five seconds was used (Carter et al., 2004; Pottier et al., 2010). Participants were able to drink water at two time points during the 1-hour WCT, in which they were allotted 30-s to consume up to 16 ounces of water. The water was provided 2-min after their third rinse and 2-min after their sixth rinse. The amount of water consumed during the first trial was measured and repeated during the second trial. The mouth rinse protocol is presented in Figure 2.

#### Heart Rate Variability and Respiratory Exchange Ratio

Participants were fitted with a heart rate monitor that was placed underneath the sternum, and a one-way breathing valve, mouthpiece, and nose clip were administered. The output of the breathing valve was connected to a metabolic cart (Parvomedics TrueOne^®^ 2400, Sandy, UT). Recordings for HRV and RER were collected simultaneously in a quiet, dimly lit room to minimize external stimuli and participants were instructed to remain as still as possible while in a seated position. Two separate recordings were obtained throughout the second and the third visit; one 15-min recording taken prior to exercise (PRE) and one 30-min recording taken immediately post exercise (IPE). The recordings obtained were analyzed in 5-min segments: the PRE time point measured the last 5-min of the 15-min recording; the second recording (30-min) was divided into five POST segments at 5–10 min, 1015 min, 15–20 min, 20–25 min, and 25–30 min.

#### HRV Analysis

HRV is the quantification of the variation between R-R intervals derived from an electrocardiogram or a beat-to-beat quantifying device (Stanley et al., 2013). HRV can be evaluated using the root mean square of successive N-N differences (RMSSD), which is a widely accepted marker of autonomic activity (Heffernan et al., 2006; Stanley et al., 2013). Analysis was completed through the Kubios HRV Software (Kubios, V 2.2, Joensuu, Finland).

#### Blood Samples and Storage

Blood samples were collected via venipuncture to determine concentrations of E, NE, INS, and FFA at pre-exercise (PRE), immediately post-exercise (IPE), and 30-min post exercise (30-P). The samples were collected in 6-mL heparinized tubes and immediately centrifuged at 2,500 rpm for 15 min. The samples were then aliquoted and stored at -80^°^ until the assays were performed. Plasma concentrations of E and NE (Abnova KA3768), INS (Alpco Ultrasensitive Insulin) and FFA (Bioassay Systems EFFA-100) were assayed using ELISA kits. Plasma volume shifts that occurred with the exercise trial were accounted for by normalizing all samples based on the established protocols of Dill and Costill (1974). Measurements of GLU (Contour Next Link, Mishawaka, IN), LA- (Lactate Plus, Nova Biomedical, Waltham, MA), hematocrit and hemoglobin (Alere Hempoint H2, Waltham, MA) were obtained via capillary puncture.

### Statistical Analyses

All data were analyzed with SPSS 24.0 (Chicago, Illinois). A Shapiro-Wilk test was performed to determine whether the HRV data set followed a normal distribution. If a violation of normality occurred, a natural logarithmic transformation was performed on RMSSD, reported as lnRMSSD (Kliszczewicz et al., 2016; Parekh and Lee, 2005). A series of trial x time repeated measures analysis of variance (ANOVA) were performed to compare the differences in the HR, lnRMSSD, RER, E, NE, INS, GLU, FFA, and LA- mean values.

Where violations of the sphericity assumption occurred, the Greenhouse-Geisser correction was used to adjust degrees of freedom. In the event of a significant F-ratio, main effects were further assessed by Bonferroni post-hoc analysis. Performance measures from both WCTs were analyzed using a paired samples *t*-test. Effect size (ES) of the mean differences was determined using Cohen’s d and the magnitude of the ES was determined by the Hopkin’s scale (Hopkins et al., 2009). A criterion alpha level of *p* ≤ 0.05 was used to determine statistical significance. All data are reported as mean ± standard deviation.

## Results

Of the original 20 participants, ten completed all experimental trials with pre-exercise and post-exercise HRV and RER recordings along with the venipuncture procedures. Two participants were excluded due to their failure to meet the cardiorespiratory fitness inclusion criteria, three were excluded due to a vasovagal response to the venipuncture procedure, one withdrew due to illness experienced outside of the study, three were excluded due to their inability to complete the protocol, and one withdrew due to a scheduling conflict.

*Performance*: Performance outcome measures can be seen in Table 2. There were no differences in time to completion of the 1-hour WCT (*p* = 0.545). Furthermore, no differences were observed in the average heart rate (*p* = 0.366), maximal heart rate (*p* = 0.324), or average pedal rate (*p* = 0.736). There was no order effect observed in the 1-hour WCT (*p* = 0.258).

**Table 2:** Performance Measures

Marker	1‐hour WCT	
	CHO	PLA
*Time to Completion (min)*	69.6 ± 6.2	68.5 ± 5.1
*Average Heart Rate (bpm)*	160 ± 18	164 ± 14
*Average Pedal Rate (RPM)*	76.8 ± 6	76.0 ± 5
*Maximum Heart Rate (bpm)*	178 ± 18	179 ± 14

*n = 10; Values presented as mean* ± *SD*.

*Significantly different between trials.

*Ratings of Perceived Exertion (RPE) and Heart Rate*: There was no main effect of mouth rinse (*p* = 0.300, *p* = 0.813) or trial x time interaction (*p* = 0.356, *p* = 0.516) for the RPE or HR. However, the RPE and HR changed over the exercise trial (*p* = 0.011, *p* = 0.002). All RPE time points after the first rinse were significantly higher in both the CHO and PLA trials (*p* < 0.05). Additionally, the HR was significantly elevated at IPE (*p* < 0.001), but returned to pre-exercise values by 30-P (*p* = 0.05).

*Heart Rate Variability*: lnRMSSD demonstrated no main effect (*p* = 0.822) or interaction effect (*p* = 0.787); however, a significant time effect occurred (*p* = 0.017). lnRMSSD was significantly reduced at IPE (*p* = 0.028) and did not return to pre-exercise values until 30-P (*p* = 0.061), indicating the reduction was due to the bout of exercise.

*Biomarkers*: There was no main effect (*p* = 0.168) or interaction effect (*p* = 0.132) for E concentrations. A significant time effect was observed (*p* < 0.001). Pairwise comparisons revealed E was elevated at IPE (*p* = 0.001) and returned to pre-exercise values by 30-P (*p* = 0.122) in both the CHO and the PLA trial, indicating the observed elevation was due to the exercise bout. There was no main effect (*p* = 0.706) or interaction effect (*p* = 0.684) for NE concentrations. A time effect was observed (*p* < 0.001) with a significant elevation occurring at IPE (*p* < 0.001) and remaining elevated at 30-P (*p* = 0.001). FFA demonstrated a significant main effect (*p* = 0.006) and time effect (*p* = 0.003); however, no interaction effect (*p* = 0.064) was observed. RMANOVA revealed that FFA were significantly elevated immediately following exercise (*p* = 0.013), but returned to pre-exercise values by 30-P (*p* = 0.238). In LA-, there was no main effect (*p* = 0.758) or interaction effect (*p* = 0.302), but a time effect (*p* < 0.001) was observed. LA- was significantly elevated at IPE (*p* < 0.001) and 30-P (*p* < 0.001) due to the bout of exercise. Insulin demonstrated no main effect (*p* = 0.706), time effect (*p* = 0.408) or interaction effect (*p* = 0.783). Furthermore, there was no main effect (*p* = 0.799), time effect (*p* = 0.384) or interaction effect (*p* = 0.113) for GLU, indicating that both INS and GLU were unaffected by supplementation. Mean values ± SD for all biomarkers are presented in Table 3.

**Table 3:** Biomarkers of ANS and Metabolic Activity Markers of plasma catecholamines are represented as epinephrine (E) and norepinephrine (NE), Markers of metabolic activity are represented as insulin (INS), glucose (GLU), lactate (LA^-^), and free fatty acids (FFA).

Marker	PLA	*p*	Effect Size	CHO	*p*	Effect Size	Trial *p*	Effect Size
*E (pg·mL^-1^)*								
PRE	62.7 ± 19.1	---	---	57.4 ± 15.9	---	---	0.339	0.149
IPE	143.8 ± 27.6*	0.000	0.863	176.1 ± 63.5*	0.000	0.788		
30-P	72.3 ± 24.7	1.000	0.212	84.1 ± 14.6	0.022	0.658		
*NE (pg·mL^-1^)*								
PRE	589.2 ± 175.1	---	---	659.5 ± 212.3	---	---	0.117	0.178
IPE	1932.5 ± 597.6*	0.000	0.836	1912.4 ± 340.1*	0.000	0.911		
30-P	1142.8 ± 476.9*	0.011	0.610	1242.7 ± 416.0*	0.001	0.619		
*INS(μIU/mL)*								
PRE	41.1 ± 19.1	---	---	38.2 ± 16.2	---	---	0.421	0.082
IPE	44.5 ± 31.4	1.000	0.065	47.9 ± 25.3	0.823	0.223		
30-P	46.9 ± 33.3	1.000	0.106	41.8 ± 25.5	1.000	0.084		
*GLU (mmol·L)*								
PRE	5.6 ± 0.3	---	---	5.5 ± 0.3	---	---	0.356	0.164
IPE	5.1 ± 0.4	0.021	0.491	5.4 ± 0.7	0.657	0.087		
30-P	5.5 ± 0.5	0.779	0.055	5.4 ± 0.7	0.516	0.091		
LA^-^ *(mmol·L)*								
PRE	0.85 ± 0.48	---	---	0.71 ± 0.35	---	---	0.143	0.164
IPE	3.88 ± 2.13*	0.004	0.700	3.70 ± 1.91*	0.001	0.737		
30-P	1.21 ± 0.23	0.080	0.431	1.27 ± 0.49	0.000	0.549		
*FFA (μM)*								
PRE	111.5 ± 26.9#	---	---	102.9 ± 54.2#	---	---	0.547	0.099
IPE	175.6 ± 39.4*#	0.000	0.687	312.2 ± 132.9*#	0.005	0.718		
30-P	115.9 ± 31.7#	0.733	0.075	212.1 ± 156.5#	0.062	0.423		

*All data are presented as Means ± SD. Trial p indicates statistical comparison between PRE values of PLA and CHO*.

* *Indicates significantly different from PRE time point. # Indicates a significant main effect. PRE = before exercise; IPE = immediately after exercise; 30-P = 30-minutes after cessation of exercise*.

*Respiratory Exchange Ratio*: There was no main effect (*p* = 0.053) or interaction effect (*p* = 0.219) observed in RER values; however, a time effect did occur (*p* = 0.004). RER was significantly lower at the post 15–20 min time point when compared to pre-exercise values.

## Discussion

The primary purpose of this study was to examine the effect of CHO rinsing on physiological responses during the post-exercise recovery period, in order to determine whether CHO rinsing could be a potential strategy to augment recovery. Additionally, we sought to quantify the downstream metabolic and autonomic responses in an effort to better understand physiological alterations in response to the proposed central effect of the CHO rinse. Overall, the findings of the study displayed no enhancement in exercise performance during a 1-hour cycling WCT with the CHO rinse. In addition to having no impact on performance, various markers of autonomic and metabolic recovery demonstrated no alterations between the PLA and CHO trials. Our original hypothesis was not supported as biomarkers of parasympathetic responses were unchanged as a result of the CHO rinse. Plasma epinephrine concentrations and HRV indicated that post-exercise autonomic recovery (return to parasympathetic tone of the resting state) was not improved with the use of the CHO rinse. Furthermore, downstream metabolic biomarkers were unaltered with the use of a CHO rinse, with no differences between trials observed in the respiratory exchange ratio, free fatty acids, insulin, glucose, and lactate.

Initial research in CHO rinsing has focused primarily on performance with several studies showing 2.0-3.7% improvements in time to completion of a 1-hour WCT (Carter et al., 2004; Chambers et al., 2009; Pottier et al., 2010). The results of the current study did not indicate a statistically significant improvement in time to completion with the use of the CHO rinse. The overall mean differences showed an approximate 1.6% improvement (~ 1-min) with the use of the rinse. When examined individually, six of the ten participants completed the WCT more quickly with the use of the CHO rinse. Rollo et al. (2010) proposed that some individuals may respond to the CHO solution, leading to performance improvements, while others may not (i.e., responders vs. non-responders). Previous studies demonstrating significant performance improvements utilized a sample with a greater mean aerobic capacity, leading to our theory that higher VO2max could be a potential factor in the individuals’ ability to respond to the supplement. In further support of our findings, the sample within the study conducted by Kulaksiz et al. (2016) consisted of recreationally active individuals, while many studies demonstrating performance improvements with a CHO rinse were conducted in trained cyclists with a higher mean aerobic capacity (Carter et al., 2004; Chambers et al., 2009; Lane et al., 2012). Future research should seek to identify which factors may lead to an individual being classified as a responder or a non-responder to a CHO rinse to better determine which athletes will benefit from rinsing and enhance sports nutrition strategies.

Delayed parasympathetic recovery is an essential consideration for athletes, due to the relationship of decreased vagal activity and diminished exercise performance (Gifford et al., 2018). Autonomic recovery can be measured non-invasively through HRV parasympathetic specific markers and circulating sympathetic neurohormones E and NE (Buchheit et al., 2007; Stanley et al., 2013). The results of the current study demonstrated no improvements in lnRMSSD recovery following the use of a CHO rinse, leading to the rejection of the hypothesis that PNS rebound would result in improved recovery from exercise. Previous research has examined the response of HRV indices following the ingestion of a CHO solution and exhibited improvements in post-exercise recovery (Moreno et al., 2013); however, this response was not observed in the current study, suggesting that the ingestion of carbohydrate may be necessary to elicit changes in HRV. Furthermore, it is well established that fluctuations in autonomic activity result in alterations in the mobilization of FFA (Wolfe et al., 1990). Importantly, with the findings of the current study demonstrating no statistical differences in autonomic activity and subsequent circulating catecholamine concentrations, it is unsurprising that the mobilization of FFA was not different between trials

When evaluating the metabolic biomarker of blood glucose, there was no effect of the CHO rinse observed. Previous literature has explored the blood glucose response to CHO rinsing during exercise, none of which have demonstrated significant changes throughout the duration of the bout (Ispoglou et al., 2015; Pottier et al., 2010). For instance, Pottier et al. (2010) examined blood glucose throughout a 1-hour cycling WCT with the use of a 6.0% CHO solution and observed no changes in blood glucose concentrations throughout the exercise. Though we did not examine glucose during the exercise trial, our findings are suggestively in agreement with those of Pottier et al. (2010), because we did not see any changes between pre- and post-exercise values with the CHO rinse. In further support of the findings of the current study, Ispoglou et al. (2015) examined glucose concentrations during a 1-hour cycling WCT with 4%, 6%, and 8% CHO solutions and saw no differences in percent change or mean concentrations of plasma glucose. This is indicative that performance improvements observed in previous research studies are unlikely to be the result of alterations in glucose concentrations.

Insulin is an important biomarker of metabolism involved in glucose regulation that has yet to be evaluated during the post-CHO rinse recovery period (James et al., 2017; Just et al., 2008; Rollo et al., 2008). The studies which have examined the insulin response to CHO rinsing have done so under resting conditions or during the exercise protocol. Just et al. (2008) observed a significant elevation in insulin concentrations for five minutes following taste stimulation with sucrose in a rested state. In contrast to those findings, a study by Rollo et al. (2008) examined changes in plasma insulin and saw no differences among insulin concentrations following the rinsing of a 6.4% CHO solution over a one-hour period. Our findings are consistent with those of Rollo et al. (2008) with no differences detected between the conditions.

Although the current study was carefully designed and implemented, it is not without limitations. The sample size resulted in the study being underpowered due to the difficulty in recruiting participants who met the inclusion criteria and were able to complete the entire protocol. Furthermore, in an effort to minimize the time commitment of participants we limited the number of trials, although the study would have benefitted from four trials: control (no water), water, CHO rinse, and PLA. Future research should implement all four trials to identify the potential physiological impact of the use of the CHO rinse.

The findings of the current study demonstrated no statistically significant performance improvements in time-to-completion of the cycling bout. However, in order to further examine the potential of responders vs. non-responders, future directions of research in CHO rinsing should examine individuals with various levels of VO2max. Additionally, no statistical differences in autonomic or metabolic function were observed between the CHO rinse and the placebo trial, resulting in the rejection of our original hypothesis. As a result, the use of a CHO rinse to improve post-exercise autonomic recovery does not appear to be a beneficial sports nutrition strategy.
